# Experience of cohort studies in Trivandrum

**DOI:** 10.1186/1753-6561-7-S5-O8

**Published:** 2013-08-30

**Authors:** K Ramadas

**Affiliations:** 1 Regional Cancer Centre, Trivandrum, India

## 

This is the results of Trivandrum Oral Cancer Screening study (TOCS) initiated in 1996 and the Trivandrum Breast Cancer Screening study (TBCS) initiated in 2006. The objective of TOC study is to evaluate the efficacy of oral cancer screening by visual inspection of the oral cavity in detecting early stages of oral cancer and in reducing mortality. Control and intervention groups have been followed up for over 16 years. Visual screening for oral cancer was found to have acceptable sensitivity and specificity, associated with detection in early stages, associated with 34% reduction in oral cancer mortality, highly cost-effective and can be readily integrated into routine care.

The TBCS study had the objective of evaluating the efficacy of breast cancer screening to reduce incidence of advanced breast cancer, increase survival and reduce mortality due to breast cancer. The intervention group received health education, regular clinical breast examination and early access to diagnostic procedures. Early stage cases, node negative cases and those who underwent conservative surgery were more among the intervention group.

